# Validating a self-report measure of HIV viral suppression: an analysis of linked questionnaire and clinical data from the Canadian HIV Women’s Sexual and Reproductive Health Cohort Study

**DOI:** 10.1186/s13104-017-2453-8

**Published:** 2017-03-24

**Authors:** Allison Carter, Alexandra de Pokomandy, Mona Loutfy, Erin Ding, Paul Sereda, Kath Webster, Valerie Nicholson, Kerrigan Beaver, Robert S. Hogg, Angela Kaida, On Behalf of the CHIWOS Research Team, Aranka Anema, Denise Becker, Lori Brotto, Allison Carter, Claudette Cardinal, Guillaume Colley, Erin Ding, Janice Duddy, Nada Gataric, Robert S. Hogg, Terry Howard, Shahab Jabbari, Evin Jones, Mary Kestler, Andrea Langlois, Viviane Lima, Elisa Lloyd-Smith, Melissa Medjuck, Cari Miller, Deborah Money, Valerie Nicholson, Gina Ogilvie, Sophie Patterson, Neora Pick, Eric Roth, Kate Salters, Margarite Sanchez, Jacquie Sas, Paul Sereda, Marcie Summers, Christina Tom, Clara Wang, Kath Webster, Wendy Zhang, Rahma Abdul-Noor, Jonathan Angel, Fatimatou Barry, Greta Bauer, Kerrigan Beaver, Anita Benoit, Breklyn Bertozzi, Sheila Borton, Tammy Bourque, Jason Brophy, Ann Burchell, Allison Carlson, Lynne Cioppa, Jeffrey Cohen, Tracey Conway, Curtis Cooper, Jasmine Cotnam, Janette Cousineau, Marisol Desbiens, Annette Fraleigh, Brenda Gagnier, Claudine Gasingirwa, Saara Greene, Trevor Hart, Shazia Islam, Charu Kaushic, Logan Kennedy, Desiree Kerr, Maxime Kiboyogo, Gladys Kwaramba, Lynne Leonard, Johanna Lewis, Carmen Logie, Shari Margolese, Marvelous Muchenje, Mary Ndung’u, Kelly O’Brien, Charlene Ouellette, Jeff Powis, Corinna Q uan, Janet Raboud, Anita Rachlis, Edward Ralph, Sean Rourke, Sergio Rueda, Roger Sandre, Fiona Smaill, Stephanie Smith, Tsitsi Tigere, Wangari Tharao, Sharon Walmsley, Wendy Wobeser, Jessica Yee, Mark Yudin, Dada Mamvula Bakombo, Jean-Guy Baril, Nora Butler Burke, Pierrette Clément, Janice Dayle, Danièle Dubuc, Mylène Fernet, Danielle Groleau, Aurélie Hot, Marina Klein, Carrie Martin, Lyne Massie, Brigitte Ménard, Nadia O’Brien, Joanne Otis, Doris Peltier, Alie Pierre, Karène Proulx-Boucher, Danielle Rouleau, Édénia Savoie, Cécile Tremblay, Benoit Trottier, Sylvie Trottier, Christos Tsoukas, Jacqueline Gahagan, Catherine Hankins, Renee Masching, Susanna Ogunnaike-Cooke

**Affiliations:** 1 Faculty of Health Sciences, Simon Fraser University, Burnaby, BC Canada; 2 Epidemiology and Population Health Program, British Columbia Centre for Excellence in HIV/AIDS, Vancouver, BC Canada; 3 Chronic Viral Illness Service, McGill University Health Centre, Montreal, QC Canada; 4 Department of Family Medicine, McGill University, Montreal, QC Canada; 5 Women’s College Research Institute, Women’s College Hospital, Toronto, ON Canada; 6 Faculty of Medicine, University of Toronto, Toronto, ON Canada

**Keywords:** Women, HIV, Viral suppression, Validation, Canada, CHIWOS

## Abstract

**Background:**

We assessed the validity of a self-report measure of undetectable viral load (VL) among women with HIV in British Columbia (BC), Canada. Questionnaire data from the Canadian HIV Women’s Sexual and Reproductive Health Cohort Study was linked with population-based clinical data from the BC Centre for Excellence in HIV/AIDS. Self-reported undetectable VL was assessed by the question: “What was your most recent VL, undetectable (i.e. <50 copies/mL) or detectable (i.e. ≥50 copies/mL)?” Laboratory measurements of VL <50 copies/mL (closest to/before study visit) were the criterion for validity analyses. We measured positive and negative predictive values (PPV, NPV) and likelihood ratios (LR+, LR−).

**Results:**

Of 356 participants, 99% were linked to clinical data. Those unlinked (n = 1), missing self-report VL (n = 18), or missing self-report and laboratory VL (n = 1) were excluded. Among the remaining 336: median age was 44 (IQR 37–51); 96% identified as cis-gender; 84% identified as heterosexual; and 45% identified as Indigenous, 40% White, 8% African, Caribbean, or Black, and 8% other/multiple ethnicities. Overall, 85% self-reported having an undetectable VL while 82% had clinical data indicating viral suppression. The PPV was 93.7 (95% CI 90.2–96.2) indicating that 94% of women who self-reported being undetectable truly were. The NPV was 80.4 (95% CI 66.9–90.2). LR+ was 3.2 (2.1–4.6) and LR− was 0.05 (0.03–0.10).

**Conclusions:**

Our self-report measure assessing undetectable VL strongly predicted true viral suppression among Canadian women with HIV. This measure can be used in research settings without laboratory data in regions with high rates of VL testing and suppression.

## Background

Combination antiretroviral therapy (cART) is now recommended for all people living with HIV regardless of CD4 cell count [1]. The clinical benefit of early initiation of cART was established in two recent randomized controlled trials, START [2] and Temprano [3], which reported reduced risks of HIV morbidity and mortality. Sustained use of and adherence to cART also has the additional benefit of lowering the risk of HIV transmission to zero by suppressing plasma viral load (VL) [4–9].

The efficacy of using treatment for individual health and for population-level prevention has been demonstrated in jurisdictions around the world including British Columbia (BC), Canada, where “Treatment as Prevention” (TasP) was formally adopted into policy in 2010 [10]. This has resulted in new global targets for reducing the burden of HIV worldwide. According to United Nations three-part “90–90–90” strategy, the goal is to have, by 2020, 90% of all people living with HIV diagnosed, 90% of all people diagnosed with HIV on cART, and 90% of all people on cART virally suppressed [11].

To monitor and evaluate progress on 90–90–90 goals, accurate assessment of viral suppression is essential. Laboratory technologies of plasma samples are the gold standard for measuring VL in clinical practice [12]. However, in the absence of linkage to clinical data, observational studies must rely on participant self-report of VL and it is unclear whether this is a valid method of assessment. In this analysis, we investigated the validity of using a brief survey question capturing most recent VL as a way to estimate viral suppression among a cohort of women living with HIV in BC, using linked questionnaire and clinical data from the Canadian HIV Women’s Sexual and Reproductive Health Cohort Study (CHIWOS).

## Methods

### The study

CHIWOS is a national, multi-site, longitudinal, community-based research study conducted *by, with*, and *for* women living with HIV, in collaboration with researchers, clinicians, service providers, policy-makers and other stakeholders (described in detail elsewhere: [13]). In total, 1425 self-identified women living with HIV (≥16 years) were enrolled into the study from three Canadian provinces: BC (n = 356), Ontario (n = 713), and Québec (n = 356).

### Procedures

Participants completed structured questionnaires at baseline, administered by Peer Research Associates (women living with HIV who have received training in community-based research) [14]. Study visits were 1.5 to 2.5 h long (median: 120 min, IQR 90–150) and took place in various community settings such as HIV clinics, AIDS Service Organizations, women’s homes, or via phone/Skype [15]. Questionnaires were web-based, developed using FluidSurveys™ software and designed to collect nationally consistent self-reported data on socio-demographics, use of HIV clinical and support services, and health outcomes including most recent VL. In BC, linkage to clinical data was possible through the Drug Treatment Program (DTP) of the BC Centre for Excellence in HIV/AIDS, a population-based registry capturing 100% of laboratory-confirmed VL data for all people living with HIV and receiving cART in BC. Linkage will be endeavored in other study provinces via the Canadian HIV Cohort (CANOC) Collaborative Research Centre [16], although incomplete linkage is expected due to varying study eligibility criteria and the absence of a single, coordinated population-based registry in Ontario and Québec.

### Final sample for this analysis

For this analysis, we used baseline questionnaire data from participants enrolled in CHIWOS in BC between August 27, 2013 and May 1, 2015, and linked clinical data from the BC Centre for Excellence in HIV/AIDS. Of 356 participants, 99% were linked to the DTP clinical database (n = 354). Those who remained unlinked (n = 1), who provided invalid responses (i.e., *“don’t know”* or *“prefer not to answer”*) to the survey question assessing VL or self-reported never receiving HIV medical care or VL results (n = 18), or who were missing both self-reported and laboratory VL (n = 1) were excluded from the analysis, leading to a final analytic sample of 336.

### Main outcome

In the CHIWOS questionnaire, self-reported VL was assessed by the following survey question: *“What was your most recent viral load, undetectable (i.e. below 50 copies/mL) or detectable (i.e. 50 copies/mL or more)?”* Criteria for validity analyses were corresponding laboratory measurements of VL <50 copies/mL, with tests closest to and before the study visit selected for analysis. For those included in analyses (N = 336), median time between study visit and most recent VL lab result was 50.5 days (IQR 20.5–87).

### Statistical analyses

Baseline characteristics of the sample were estimated. Following this, positive and negative predictive values (PPV, NPV) and likelihood ratios (LR+, LR−) were computed for the outcome, overall and stratified by select socio-demographic variables including ethnicity (Indigenous *vs.* White *vs.* African, Caribbean, or Black vs. other/multiple ethnicities), education (<High school vs. ≥High school), and illicit drug use within the past 3 months (Yes vs. No). PPV is the probability that participants who self-report being “undetectable (i.e., below 50 copies/mL)” truly have laboratory measurements of VL <50 copies/mL, while NPV is the probability that participants who self-report being “detectable (i.e., above 50 copies/mL)” truly have laboratory measurements of VL ≥50 copies/mL [17]. LR+ (sensitivity/1-specificity) corresponds to the clinical concept of “ruling-in disease”, and indicates how much a “positive test” (in this case, self-reported undetectable VL) *increases* the odds that a patient actually has the health outcome (in this case, true suppression) [18]. LR− (1-sensitivity/specificity) corresponds to “ruling-out disease”, and indicates how much a “negative test” (in this case, self-reported detectable VL) *decreases* the odds that a patient has the health outcome (in this case, true suppression) [18]. LRs greater than 1 indicate that true suppression is likely (the larger the number, the more likely suppression), while LRs between 0 and 1 indicate that suppression is unlikely (the closer the number to zero, the less likely suppression) [18]. Analyses were conducted using SAS version 9.4 (SAS, North Carolina, United States).

## Ethical statement

All Principal Investigators’ institutional Research Ethics Boards granted ethical approval including Women’s College Hospital, McGill University Health Centre, and in BC, Simon Fraser University and the University of British Columbia/Providence Health (ethical approval numbers: 2012s0959 and H12-03326). Ethical approval was also sought and granted from hospitals and AIDS Service Organizations if recruitment occurred there. All participants provided consent (written or oral in the case of phone interviews) to participate.

## Results

### Baseline characteristics of the sample

Among the 336 women included in this analysis, 99% self-reported accessing HIV medical care in the past year and 96% self-reported currently taking cART. Overall, 85% (n = 286) self-reported having undetectable VL and 82% (n = 276) had laboratory data indicating VL <50 copies/mL.

Baseline characteristics of the sample are provided in Table 1. Median age was 44 (IQR 37–51). The majority of women identified as cis-gender (96%) and heterosexual (84%). Forty-five percent identified as Indigenous and 40% as White; a small proportion also identified as African, Caribbean, or Black (8%), or other/multiple ethnicities (8%). Most completed at least high school (73%) yet reported low annual household incomes of <$20,000 (72%). Thirty-four percent reported illicit drug use in the past 3 months, and 9% were incarcerated in the past year.

**Table 1:** Baseline characteristics of women with HIV enrolled in British Columbia in the CHIWOS Study (n = 336)

	n (%)
Clinical variables	
Self-reported HIV medical care in past year (yes)	333 (99)
Self-reported current cART use (yes)	323 (96)
Self-reported undetectable VL (yes)	286 (85)
Laboratory-confirmed VL <50 copies/mL (yes)	276 (82)
Socio-demographic variables	
Age, median (IQR)	44 (IQR 37–51)
Gender identity	
Cis gender	323 (96)
Trans and gender diverse women	13 (4)
Sexual orientation	
Heterosexual	281 (84)
LGBTQ	55 (16)
Ethnicity	
Indigenous	151 (45)
White	133 (40)
African/Caribbean/Black	26 (8)
Other ethnicities	26 (8)
≥High school education (yes)	245 (73)
<$20,000 annual household income (yes)	243 (72)
Illicit drug use in past 3 months (yes)	113 (34)

*VL* viral load, *cART* combination antiretroviral therapy, *LGBTQ* lesbian/gay/bisexual/two-spirited/queer, *CHIWOS* Canadian HIV Women’s Sexual and Reproductive Health Study

### Validity analyses of viral load data taken from questionnaires and laboratory results

Results of validity analyses are provided in Tables 2 (overall sample) and 3 (sub-populations). For the overall sample, the PPV was 93.7 (95% CI 90.2–96.2), indicating that 93.7% of women who self-reported being undetectable via questionnaire truly were virally suppressed according to laboratory results. The NPV was 80.4 (95% CI 66.9–90.2), suggesting that 80.4% of women who self-reported being detectable truly were not virally suppressed according to laboratory results. The LR+ was 3.2 (95% CI 2.1–4.6), indicating that self-reporting undetectable offers a moderate increase in the likelihood of true suppression. The LR− was 0.05 (95% CI 0.03–0.10), suggesting that reporting a detectable VL provides a large and conclusive decrease in the likelihood of true suppression. In terms of specific sub-populations, no significant differences in PPVs were observed, with point estimates ranging from 90.4 to 96.0 (and overlapping confidence intervals) across select socio-demographic variables including ethnicity, education, and recent illicit drug use. Some variations in point estimates, though, were seen in NPVs. For instance, those who had completed high school had a higher NPV [84.4 (95% CI 67.2–94.7)] compared with those who did not [73.7 (95% CI 48.8–90.9)]; while the confidence intervals were large and overlapped owing to small cell sizes and low power, the differing effect estimates suggest education level may be a marker for accurate reporting of detectable VL. Illicit drug use was not a marker, however. The values of likelihood ratios (LR+ and LR−) were similar to those found with the overall sample.

**Table 2:** Predictive values and likelihood ratios of self-reported undetectable VL in overall CHIWOS cohort (n = 336)

			PPV (95% CI)	NPV (95% CI)	LR+ (95% CI)	LR− (95% CI)
	Laboratory-confirmed VL (from BC-CfE)^a^					
	VL <50 copies/mL	VL ≥50 copies/mL				
Self-report VL (from CHIWOS)						
Undetectable (i.e. <50 copies/mL)	267	18	93.7 (90.2–96.2)	80.4 (66.9–90.2)	3.2 (2.1–4.6)	0.05 (0.03–0.10)
Detectable (i.e. ≥50 copies/mL)	10	41				

*PPV* positive predictive value, *NPV* negative predictive value, *LR*+ positive likelihood ratio, *LR*− negative likelihood ratio, *95% CI* 95 percent confidence interval, *VL* viral load, *BC*-*CfE* BC Centre for Excellence in HIV/AIDS, *CHIWOS* Canadian HIV Women’s Sexual and Reproductive Health Cohort Study

^a^Gold standard (True diagnosis)

**Table 3:** Predictive values and likelihood ratios of self-reported undetectable VL in CHIWOS sub-populations (n = 336)

	PPV (95% CI)	NPV (95% CI)	LR+ (95% CI)	LR− (95% CI)
Ethnicity				
Indigenous	92.6 (86.4–96.5)	86.7 (69.3–96.2)	3.8 (2.1–6.6)	0.05 (0.02–0.12)
Caucasian	94.8 (89.1–98.1)	70.6 (44.0–89.7)	2.9 (1.5–5.5)	0.07 (0.03–0.16)
African/Caribbean/Black	91.3 (72.0–98.9)	100.0 (29.2–100.0)	2.5 (0.8–7.3)	NA
Other	96.0 (79.6–99.9)	0 (0–97.5)	1.0 (0.9–1.0)	NA
Education				
<High school	91.4 (82.3–96.8)	73.7 (48.8–90.9)	3.1 (1.6–6.1)	0.10 (0.04–0.25)
≥High school	91.4 (82.3–96.8)	84.4 (67.2–94.7)	3.4 (2.1–5.6)	0.03 (0.01–0.08)
Illicit drug use in past 3 months				
Yes	90.4 (81.9–95.8)	80.0 (61.4–92.3)	3.7 (2.0–6.8)	0.10 (0.04–0.22)
No	95.0 (91.0–97.6)	80.1 (58.1–94.6)	2.6 (1.6–4.3)	0.03 (0.01–0.09)

*PPV* positive predictive value, *NPV* negative predictive value, *LR*+ positive likelihood ratio, *LR*− negative likelihood ratio, *95% CI* 95 percent confidence interval, *VL* viral load, *CHIWOS* Canadian HIV Women’s Sexual and Reproductive Health Cohort Study

## Discussion

Our findings demonstrate that a brief survey question measuring self-reported undetectable VL strongly predicted true viral suppression, as confirmed via laboratory results, among a cohort of women living with HIV in BC, Canada. The PPV was high (>90) and the LR+ greater than 1, both for the overall sample and for specific sub-populations, suggesting that this self-report measure is a valid method of assessment of undetectable VL among diverse communities of women living with HIV where VL testing is common and the true prevalence of viral suppression is high (i.e., 82% were undetectable as measured through laboratory data). The NPV was lower in comparison (ranging from 70 to 100 depending on the demographic group) but the LR− was extremely close to zero, suggesting that for participants who reported being detectable, we can have high confidence that this self-report is accurate and the participant is *not* suppressed.

Jurisdictions around the world are currently implementing policy and programmatic interventions to meet the United Nation’s 90–90–90 targets, which aim to have 73% of all people living with HIV virally suppressed by 2020 [11]. This goal has both individual and public health value, with HIV viral suppression achieved through early and sustained use of cART consistently shown to be associated with reduced disease progression, extended life expectancy, and zero risk of onward transmission [2–9]. Measuring progress on achieving suppression in research settings is important but may be challenged by a lack of clinical data. We experienced this in CHIWOS, where only one of our three study provinces currently has laboratory data available through linkage with a population-based clinical registry.

As shown in this study, 82% of women living with HIV in BC had an undetectable VL, defined in this study as below 50 copies/mL, which exceeds the United Nation’s 90–90–90 targets. Our threshold for designating a VL as undetectable was stricter than some studies (i.e., that use 300 to 500 copies/mL [19]), but this cut-off is consistent with past reporting in Canada [20, 21], and even with this conservative measure, the prevalence of HIV suppression was high and comparable to observational data from other cohorts of women living with HIV [22, 23]. Moreover, based on the positive and negative predictive values and likelihood ratios, the majority of women were able to accurately report their VL. This self-reported measure can, therefore, be used in research settings without laboratory data confirmation and where VL testing and the true prevalence of viral suppression is high to assess 90–90–90-related goals.

There are limitations to this study. We were unable to validate this measure across all study provinces and women in different regions do differ in health and social characteristics. For instance, a much larger proportion of women identify as African, Caribbean or Black in Ontario (32%) and Québec (46%) compared with BC (8%) where more women identify as Indigenous (45%) and have histories of injection drug use (BC—63% vs. Ontario—19% vs. Québec—23%) (data not shown). Women in Ontario are also younger, with a median age of 41 (IQR 34–49) versus 44 in BC (IQR 37–51) and 46 in Québec (IQR 38–53), and are less likely to be currently on cART (BC–89% vs. Ontario—75% vs. Québec—92%) with a self-reported undetectable VL (BC—80% vs. Ontario—71% vs. Québec—87%) (data not shown). However, the current analysis demonstrated high PPVs for the overall sample and specific sub-populations including women of varying ethnicities and drug use histories. The range in NPVs was lower and wider depending on demographics characteristics; thus, confidence in the true negative rate may vary by key factors such as education as shown in this analysis.

Another limitation is that we notified women during the screening process that we would be asking for their clinical information. Thus, the high validity here is likely not just a result of women knowing their VL but also because some women accessed this information from their clinic before the actual study visit. This is perhaps particularly true for women interviewed in a clinic setting. To examine this, we ran a sub-analysis among women who self-reported that their primary site of HIV medical care was Oak Tree Clinic [n = 132 (39% of sample)], one-third of whom were interviewed onsite [n = 41 (31%)], versus women who did not access this clinic. The PPVs for these two groups were similar [Oak Tree Clinic: 95.4 (95% CI 89.6–98.5) vs. Non-Oak Tree Clinic: 92.6 (85% CI 87.6–96.0)]. However, the NPVs were much higher for Oak Tree Clinic patients [91.3 (95% CI 72.0–98.9)] compared with non-Oak Tree Clinic patients [71.4 (95% CI 51.3–86.8)]. Thus, while prompting participants for clinical information does not appear to have altered the proportion of those undetectable that are able to correctly identify as such, likely given the high VL suppression rates to begin with, these data do suggest this screening procedure may have impacted accurate reporting of a detectable VL.

Finally, we also conducted a sub-analysis to determine whether the 18 participants who were unable to self-report their VL (either because they reported *“don’t know”*, *“prefer not to answer”*, or *“never received care/VL results”*) and thus excluded from this analysis were more, less, or equally likely to be undetectable than those who were included. Among these participants, 56% (n = 10) were detectable. This compares to only 18% (n = 59) of those were included in this analysis. It is possible that these women did not feel comfortable sharing this personal information or were simply unaware owing to competing life demands preventing access to and engagement in care. All of these limitations are important to consider in research assessing VL levels.

Engagement in treatment, care, and support is critical to sustained viral suppression, and, in turn, improved individual health and HIV prevention [4–9]. Future research will be conducted by our team to assess the prevalence and predictors of HIV clinical outcomes in this cohort, the largest study of women living with HIV in Canada.

## Abbreviations

VL: viral load
cART: combination antiretroviral therapy
TasP: treatment as prevention
PPV: positive predictive value
NPV: negative predictive value
LR+: positive likelihood ratio
LR−: negative likelihood ratio
BC: British Columbia
CHIWOS: Canadian HIV Women’s Sexual and Reproductive Health Cohort Study
